# Finding the direction of lowest resilience in multivariate complex systems

**DOI:** 10.1098/rsif.2019.0629

**Published:** 2019-10-30

**Authors:** Els Weinans, J. Jelle Lever, Sebastian Bathiany, Rick Quax, Jordi Bascompte, Egbert H. van Nes, Marten Scheffer, Ingrid A. van de Leemput

**Affiliations:** 1 Department of Aquatic Ecology and Water Quality Management, Wageningen University, PO Box 47, 6700 AA, Wageningen, The Netherlands; 2 Department of Evolutionary Biology and Environmental Studies, University of Zurich, Winterhurerstrasse 190, 8057 Zurich, Switzerland; 3 Computational Science Lab, University of Amsterdam, 1098 XH Amsterdam, The Netherlands

**Keywords:** complex networks, resilience, stability

## Abstract

The dynamics of complex systems, such as ecosystems, financial markets and the human brain, emerge from the interactions of numerous components. We often lack the knowledge to build reliable models for the behaviour of such network systems. This makes it difficult to predict potential instabilities. We show that one could use the natural fluctuations in multivariate time series to reveal network regions with particularly slow dynamics. The multidimensional slowness points to the direction of minimal resilience, in the sense that simultaneous perturbations on this set of nodes will take longest to recover. We compare an autocorrelation-based method with a variance-based method for different time-series lengths, data resolution and different noise regimes. We show that the autocorrelation-based method is less robust for short time series or time series with a low resolution but more robust for varying noise levels. This novel approach may help to identify unstable regions of multivariate systems or to distinguish safe from unsafe perturbations.

## 1. Introduction

Many complex systems are managed or structured such that they are relatively stable, in the sense that they can maintain the same functions. Examples include the human body [1], financial systems [2], ecosystems or social systems [3]. All of these systems can be represented as networks [4] with multiple interacting entities, such as organs, banks or companies, species and abiotic factors or individual human beings [5]. All network entities are continuously disturbed by external events that bring the full system somewhat out of balance. For instance, climatic extremes, diseases or human interference may result in a temporary increase or decrease in abundance of one or more species [6]. Environmental fluctuations and disturbances affect different species in different ways [7,8], and particular compounded perturbations may have much larger impacts than when such perturbations occur in isolation [9]. It is intuitively straightforward that for each system there is a particular type of perturbation (in the sense that a certain set of network entities is disturbed simultaneously in a particular way) to which the system is the most sensitive [10]. This raises the question of whether we might be able to deduce such ‘weak spots’ in the myriad of possible combinations of pressures on the system.

In this study, we are thus interested in finding the particular combinations of pressures from which a system will recover the slowest. In other words, we aim to identify network regions with low resilience, where resilience is defined as the rate at which a system recovers after a perturbation, also often called engineering resilience [11]. The underlying configuration of the network and the interactions between elements is often unknown, making it hard to rely on models to simulate dynamics and find such weak spots in the system. Another approach is to use observed time series to search for combinations of variables with low resilience. If we assume a homogeneous network where all nodes and connections are similar, methods exist that may find universal patterns of resilience [12]. However, for heterogeneous networks, other approaches are required. One line of research has looked at the rate of recovery from perturbations as an indicator of resilience. For example, if a system is intrinsically slow, this should be reflected in the observed time series by a high autocorrelation [13–15] and a high variance [16,17]. While most literature on resilience indicators (also often called ‘early warning signals’) focuses on univariate data, recently the first steps towards resilience indicators based on multivariate time series (i.e. of network-type systems) have been taken. Suggested metrics to indicate the overall resilience of the system include the autocorrelation of the projection of data on the first principal component (PC) using principal component analysis (PCA) [14], combinations of cross-correlations between system elements and variance of individual elements [18], mean autocorrelation and variance [19] and the maximum value of the covariance matrix [20] . However, so far, these studies have mostly focused on finding a scalar indicator of resilience, and not so much on identifying the combination of variables involved.

**Figure 1.**
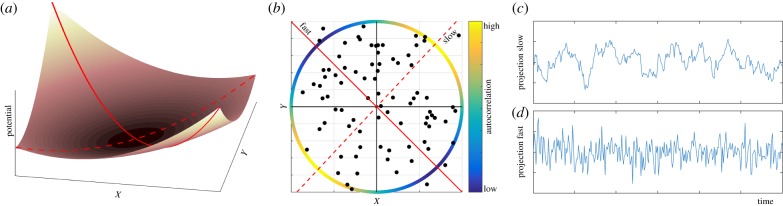
Using autocorrelation in a two-dimensional system to predict directions of fast and slow recovery. (*a*) Stability landscape of two interacting species (*X* and *Y*), showing that the speed of recovery depends on the direction of a disturbance. The speed is indicated by the slope of the stability landscape. (*b*) Autocorrelation along different directions in the system’s phase space. The scatterplot shows part of the time series. Red lines indicate the slowest direction (dashed line), i.e. with highest autocorrelation, and the fastest direction (solid line), i.e. with lowest autocorrelation. The coloured circle indicates the autocorrelation in every direction. (*c*–*d*) Projected time series on the slowest (*c*) and the fastest (*d*) direction. (Online version in colour.)

Here, we propose that one could use observed natural fluctuations to map the multivariate pattern of indicators of slowness such as temporal autocorrelation (figure 1*b*). The basic idea is most easily illustrated from a stability landscape illustration of a hypothetical two-dimensional system describing the dynamics of two interacting species *X* and *Y* (figure 1*a*). From the shape of the stability landscape, it is intuitively clear that a disturbance resulting in an increase or decrease of both species *X* and *Y* will return to equilibrium relatively quickly. By contrast, the system will recover much more slowly from a disturbance of the same strength resulting in an increase in *X* combined with a decrease in *Y* , or vice versa. Now, if we assume this system to be continuously perturbed in random directions, we can use the observed time series of *X* and *Y* to find the direction of slowest recovery simply by computing temporal autocorrelation or variance projected on all possible axes (figure 1*b*–*d*). In the two-dimensional case finding this slow direction can be done by brute computational force. However, as the number of dimensions increases it becomes impossible to scan all directions. We will show how novel ways of using known tools based on autocorrelation or variance allow scanning for the direction of lowest resilience even in highly complex networks.

We assess the suggested methods by applying them to synthetic data where we know the underlying mechanisms. Since in multivariate systems the link between a high autocorrelation or variance and a slow recovery is not as straightforward as in univariate systems, we also assess what we can expect from these resilience indicators in our multivariate examples. We pick three example models with varying degrees of complexity that allow us to compare the predictions with the actual direction of slowest recovery. Furthermore, we evaluate the robustness of both autocorrelation- and variance-based approaches for the length and resolution of the data and for different noise regimes. We introduce a test to assess if a particular real-world multivariate time series is suitable for the proposed analyses and discuss which method one should preferably use in which case.

## 2. Methods

### 2.1. Finding the direction of slow recovery

In order to find the slowest direction in a multivariate time series, we detect the direction of highest autocorrelation by using the Min/Max autocorrelation factors (MAF) analysis [21], which we explain below. Additionally, we detect the direction of highest variance by using the well-known PCA. We use simulated multivariate time series with equal temporal spacing between data points to investigate the general applicability and performance of both methods.

The MAF algorithm detects the direction of the highest variance of the first difference (difference between consecutive time points) of the time series. In a time series with high autocorrelation, the similarity between consecutive time points is high, which relates to a low variance in the first difference. Similarly, low autocorrelation relates to high variance in the first difference. The MAF algorithm detects the direction of maximum autocorrelation in a four-step process:

1. We transform the data to ensure that they have an identity matrix as the covariance matrix. In line with [22], we use an ‘SDS transform’ (spectral decomposition sphering):
  $$X_{\mathrm{SDS}}=X\;*\;U\;*\;D^{-(1/2)}\;*\;U^{\prime },$$
  where *X* is the original dataset, *X*_SDS_ is the transformed data, *U* is the eigenvector matrix of the covariance matrix of the data and *D* is a diagonal matrix with eigenvalues of the covariance matrix.
2. We calculate the first differences of *X*_SDS_, resulting in [*X*_SDS_(*t*) − *X*_SDS_(*t* + 1)].
3. We calculate the eigenvector matrix *V* and the eigenvalues *E* of the covariance matrix of [*X*_SDS_(*t*) − *X*_SDS_(*t* + 1)]. These eigenvalues can be used to determine how different the variances of the different eigenvectors are.
4. We calculate the MAFs:
  $$W_{\mathrm{MAF}}=U\;*\;D^{-(1/2)}\;*\;U^{\prime }\;*\;V.$$

More details about the procedure can be found in [21–23]. The output of the MAF analysis is a set of components called the MAFs, which are ordered from high to low autocorrelation. These can be compared with the PCs of a PCA, which are ordered from high to low variance. So like the PCs in PCA, we can project the data on the MAFs or summarize the data using only a number of MAFs to reduce the dimensionality. In contrast to PCs, the MAFs do not have to be orthogonal to each other. Since a high autocorrelation is linked to low resilience, the MAFs order the directions of the system from low to high resilience.

To be able to compare the MAFs, we use the MAF eigenvalues (*E*) belonging to the eigenvectors of the covariance matrix of [*X*_SDS_(*t*) − *X*_SDS_(*t* + 1)] that we calculated in step 3. Similar to the explained variance in PCA, the MAF eigenvalues provide a weight to the autocorrelations projected on each MAF. In contrast to PCA, a MAF with a low eigenvalue indicates that the autocorrelation of the projected time series is higher than all other directions, whereas a high eigenvalue indicates a low autocorrelation.

### 2.2. Models

To test and compare the potential methods to detect the direction of lowest resilience based on multivariate time series, we apply them to time series generated by three different models. The models have a deterministic part and a stochastic part. For the stochastic models, we use an Euler–Maruyama integration. For the deterministic models an Euler integration is used. To generate the time series, we used Grind for Matlab [24].

#### 2.2.1. Metapopulation model

First, as an example of a gradient non-reactive system, we use a classical ecological model that is known for having alternative stable states (a bistable model) [25]. Alternative stable states are multiple states that are stable under the exact same parameter settings. The model describes the abundance of a logistically growing species that is being harvested following a Holling’s type III functional response. The modelled species could for instance represent a plant that competes for space and is being grazed by herbivores. The grazing efficiency of the herbivores may increase with plant abundance until a certain biomass is reached, at which point the herbivores become saturated. For this study, we simulate a metapopulation with three patches and assume that the modelled species can migrate between the patches,

2.1 $$dN_{i}=\left[N_{i}\left(1-\frac{N_{i}}{K_{i}}\right)-\frac{c_{i}N_{i}^{2}}{1+N_{i}^{2}}+\sum_{\;j\neq i}d_{ij}(N_{\;j}-N_{i})\right]\;\text{d}t+\sigma_{N_{i}}dW_{i},$$

where *N*_*i*_ is the abundance of the species in location *i*, *K*_*i*_ is the carrying capacity at location *i*, *c*_*i*_ is the maximum harvesting rate and *d*_*ij*_ is a symmetric matrix describing migration between patch *i* and *j*. Finally, each patch is affected by noise, with *dW*_*i*_ representing a Wiener process with mean 0 and variance *σ* that is uncorrelated for the different variables. Default parameter settings are: *K*_1_ = 10, *K*_2_ = 13, *K*_3_ = 8, *c*_1_ = 3, *c*_2_ = 2, *c*_3_ = 2.3, *d*_21_ = *d*_12_ = 0.2, *d*_31_ = *d*_13_ = *d*_32_ = *d*_23_ = 0.08 and $\sigma_{N_{i}}=0.02$. A time step of 0.01 was used for integration. The parameters were chosen such that some asymmetries occur in the resilience in different directions.

It should be noted that this model is extremely simplified and the parameters are not based on observations. This first model is chosen because it is well known and can easily be used for visualizations and for explaining how to interpret the MAF results.

#### 2.2.2. Sahara model

Second, as an example of a non-gradient non-reactive system, we use a simple climate model describing vegetation–precipitation interactions in four regions of the Sahara. This model has been used to explain the shift from a vegetated state to a desert state in the Sahara region. The model was developed in [26] and made spatially explicit in [27]. The model describes the growth of the vegetation as a function of the current vegetation and the equilibrium vegetation cover, which depends on the precipitation in that location,

2.2 $$dV_{i}=\left[\frac{V^{*}(P_{i})-V_{i}}{\tau }\right]\;\text{d}t+\sigma dW_{i},$$

where *V* is the vegetation cover, V*(*P*_*i*_) is the equilibrium vegetation cover as a function of the precipitation at location *i* and *τ* is the characteristic time scale.

The vegetation equilibrium is described by

2.3 $$V^{*}(P_{i})=1.03-\frac{1.03}{1+\alpha ((P_{i}-P_{1})/\text{exp}(\gamma \delta ))},$$

where *δ* stands for the growing degree days (−900 K). The dependency of vegetation on temperature in the Sahara is, however, rather unimportant compared with rainfall. The parameter *γ* determines how steep the V*(*P*_*i*_) curve is, i.e. the sensitivity to rainfall. Precipitation reacts much faster than vegetation cover and is therefore assumed to be in its equilibrium (quasi-steady-state assumption), which depends on *V*,

2.4 $$P_{i}(V)=P_{0_{i}}+s_{i}B+\sum_{\;j=1}^{N}k_{ij}V_{\;j},$$

where $P_{0_{i}}+s_{i}B$ is the amount of precipitation if no vegetation existed and **k**_*ij*_ is the sensitivity of the precipitation in location *i* to the vegetation in location *j*. Therefore **k** is the parameter that couples the locations to each other. Default parameter settings are chosen in line with [27] as *N* = 4, *τ* = 1, $\sigma_{V_{i}}=0.02$, *α* = 0.0011, *β* = 28, *δ* = 9100, *P*_1_ = 60.6855, *P*_0_ = [ − 50, 40, 210, 40], *s* = [1.7, 0.8, 0.2, 0.9], *B* = 100 and $k=\left[\begin{array}{cccc} 243 & 30 & 50 & 50\\ 135 & 24 & 15 & 15\\ 72 & 12 & 75 & 10\\ 18 & 18 & 10 & 25 \end{array}\right]$. A time step of 0.01 was used for integration.

This Sahara model is fitted to observations and is therefore slightly more realistic than the meta-population model.

#### 2.2.3. Gene regulatory network

Third, as an example of a non-gradient reactive system, we use a simple network of gene regulations among five genes, described by Chen *et al.* [18]. This model describes the concentration of five molecules (e.g. gene or protein expressions),

2.5 $$\begin{array}{rl} & \text{d}z_{1}=\left[90|P|-1236+\frac{240-120|P|}{1+z_{3}}+\frac{1488z_{4}}{1+z_{4}}-30|P|z_{1}\right]\\ & \;\;\;\;\text{d}t+\sigma dW_{1},\\ & \text{d}z_{2}=\left[75|P|-150+\frac{60-30|P|}{1+z_{1}}+\frac{(240-120|P|)z_{3}}{1+z_{3}}-60z_{2}\right]\\ & \;\;\;\;\text{d}t+\sigma dW_{2},\\ & \text{d}z_{3}=\left[-1056+\frac{1488z_{4}}{1+z_{4}}-60z_{3}\right]\text{d}t+\sigma dW_{3},\\ & \text{d}z_{4}=\left[-600+\frac{1350z_{5}}{1+z_{5}}-100z_{4}\right]\text{d}t+\sigma dW_{4}\\ \text{and}\; & \text{d}z_{5}=\left[108+\frac{160}{1+z_{1}}+\frac{40}{1+z_{2}}+\frac{1488}{1+z_{4}}-300z_{5}\right]\text{d}t+\sigma dW_{5}, \end{array}\}$$

where *z*_*i*_ is the concentration of molecule *i* and *P* is a scalar control parameter. The gene regulation growth rates are described by the Michaelis–Menten equation and the degradation rates are proportional to the concentration of the genes. There is a stable equilibrium at $\bar{Z}=(\bar{z_{1}},\bar{z_{2}},\bar{z_{3}},\bar{z_{4}},\bar{z_{5}})=(1,0,1,3,2)$ and a tipping point at *P* = 0. For our simulations, we use *P* = 0.35 and *σ* = 0.2. A time step of 0.001 was used for integration.

This model is not based on observations, but it is tuned to display dynamics not unlike real biomarker dynamics [18]. Furthermore, it is a more complex model than the other two models. In this way, our models have different levels of realism and different levels of complexity.

### 2.3. Perturbation experiments

To verify whether the direction with the highest autocorrelation is also the direction in which perturbations recover slowest, we performed perturbation experiments in the direction of the different MAFs, using the deterministic models. We expect that the speed of recovery of perturbations in the direction of the MAF will be ordered according to the order of the MAFs. This should be true for systems that return to their equilibrium in a relatively linear way. However, when strong spiralling dynamics occur, the system can move away from the direction of the MAF after the perturbation and recovery rates may become different. Therefore, we expect that the initial recovery rate is well ordered according to the MAFs, and the later recovery rates are only well ordered when the system recovers in a linear way. We capture the initial recovery time by looking at the moment when the perturbation is at 10% of its recovery. In real-life applications, there is often an interest in more than 10% recovery. For example, an ecological system is normally not labelled as ‘recovered’ until it is indistinguishable from the situation prior to the perturbation. Therefore, recovery times are also calculated for 50% and 90% recovery. For all perturbation experiments, perturbation size is three times the standard deviation of the Gaussian white noise process used for the simulations.

Last, to check if the first MAF really provides the direction of slowest recovery, we did 1000 random perturbations for every model and calculated the recovery times for every one of them to assess if the perturbation on the first MAF was really the perturbation that would lead to the longest recovery.

### 2.4. Performance of Min/Max autocorrelation factors versus principal component analysis

We evaluated the effect of data length and resolution on the performance of MAF and PCA. To test whether the time series is of sufficient length, we performed a block bootstrap with increasing block size. We started with a block size of 0.1% of the data size, and then we randomly picked 100 blocks in the data. The blocks could overlap. For every block, we calculated the first MAF and first PC, resulting in a distribution based on 100 blocks, of which we calculated the median and the 90% confidence interval (5% and 95% boundary). Next, we increased the block size and repeated the analysis. We repeated this until the block size was 10% of the data size. If the confidence interval converges to a small value, we conclude that the data are of sufficient length. This procedure can be done with any available dataset to evaluate whether it is of sufficient length and quality for our analysis.

To examine the effect of data resolution, we again performed a block bootstrap with blocks of size 1000 for different distances between data points. We chose the blocks by starting at a random point in the time series and then taking every *n*th point until the box was full at 1000 points. We let *n* range from 1 to 1000. Again, we used 100 boxes per *n*, and we calculated the median and the 90% confidence interval.

Furthermore, we tested the performance of MAF and PCA in the case that noise is unequally distributed over the variables. For this, we used the metapopulation model, with different noise levels for each variable. We simulated all combinations of noise levels, keeping the sum of the noise ($\sum_{i=1}^{n}\sigma_{N_{i}}$) at a constant of 0.2. For every noise regime, we compared the similarity of the obtained MAF and PCA with the true direction of slowest recovery (see below).

### 2.5. Metric for comparing directions

In order to evaluate the performance of MAF and PCA, we compared both of them with the true direction of slowest recovery. We calculate the latter with the deterministic version of the model (using 50% recovery). Then, we calculate the angle between the first (MAF or PCA) component and the vector in which the system shows slowest recovery when perturbed along that vector with the formula

2.6 $$\theta =\cos^{-1}\frac{C∙V}{∥C∥\;∥V∥},$$

where *C* is the calculated direction (first MAF or first PC) and *V* is the real direction of slowest recovery. The • operator indicates the dot product. Next, we use the following probability density function that calculates the probability of finding an angle *θ* when comparing two random vectors with each other:

2.7 $$h(\theta )=\frac{1}{\sqrt{\pi }}\frac{\Gamma (d/2)}{\Gamma ((d-1)/2)}\cdot {\left(\sin\theta \right)}^{d-2},$$

where *Γ* is the Gamma function (a factorial function that can handle non-integer numbers) and *d* is the dimension of the input vectors [28].

We calculate the vector similarity as 1− *p*, where *p* is the probability of finding two random vectors that have an angle that is equal to or smaller than the angle between the two vectors (using equation (2.7)). This depends on both the angle of the two vectors and the dimensionality of the space [28].

## 3. Results

### 3.1. Interpreting Min/Max autocorrelation factors analysis

In this section, we will apply the MAF analysis to the three models and discuss the interpretation of the results of a MAF analysis. We start with the three-dimensional meta-population model, since the low dimensions allow for clear visualization.

**Figure 2.**
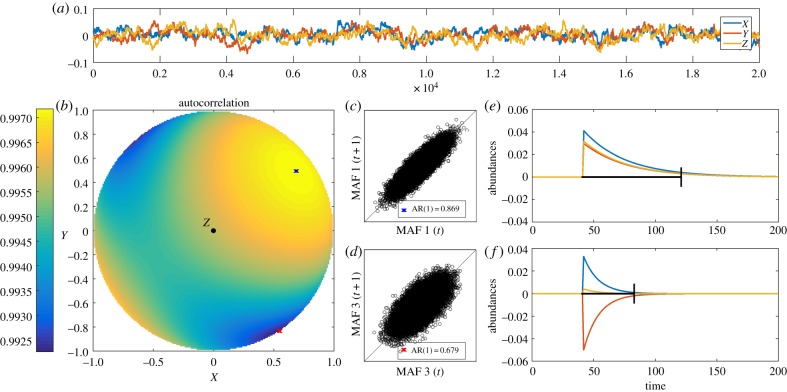
Direction of slow and fast recovery as detected by MAF. (*a*) Time series of the metapopulation model with three patches. (*b*) Autocorrelation for all possible directions in the three-dimensional plane, for *Z* > 0. The blue *X* indicates the direction of the first MAF (slowest direction), the red *X* indicates the direction of the last MAF (fastest direction). (*c*,*d*) Lag-1 autocorrelation for the projected time series on MAF 1 (*c*) and MAF 3 (*d*). (*e*,*f*) Perturbation experiments on the first and last MAF, showing that a perturbation on the first MAF results, as expected, in a slower recovery than a perturbation on the last MAF. The black lines indicate 90% recovery. (Online version in colour.)

First, we calculate the autocorrelation in all possible directions. Just like the two-dimensional example in figure 1, we depict the autocorrelation for the different directions with a colour gradient. In the two-dimensional example, we plotted it on a circle, but in this three-dimensional case we need a sphere to visualize all directions (figure 2*b*). It is important to note that, just like the circle in figure 1*b*, only half of the sphere is needed since the circle is symmetrical (e.g. autocorrelation in direction [1 1 1] is the same as autocorrelation in direction [ − 1 − 1 − 1]). Therefore, we can look at the sphere from any side. We choose to look at the side where *Z* > 0 (figure 2*b*), but any other angle would give exactly the same result. We show how the MAF analysis accurately captures the direction of highest (blue *X*) and lowest (red *X*) autocorrelation in this case (figure 2*b*). Figure 2*c*,*d* indicates how the autocorrelation differs in the two directions. Perturbations in the direction of the first and last MAF show strong differences in recovery time (figure 2*e*,*f*), where a perturbation on the first MAF (figure 2*e*) lasts longer than a perturbation on the last MAF (figure 2*f*).

**Figure 3.**
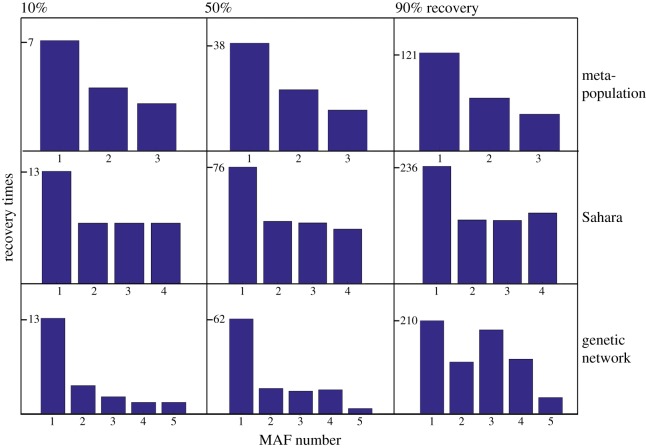
Recovery times for 10% (left), 50% (middle) or 90% (right) recovery. The initial recovery (10%, left three barplots) is well ordered by MAF, with low MAF numbers corresponding to high recovery time; 50% recovery is well ordered for the meta-population and the Sahara model; and 90% recovery is only well ordered for the meta-population model, which responds in a gradient way. However, in all cases, the recovery time is highest for the first MAF, even for the nonlinear genetic network. (Online version in colour.)

Next, we perform the MAF analysis for the other two models. After obtaining the MAFs, we perturb the system on the different MAFs. The expectation is that the perturbation on the first MAF, the one with the highest autocorrelation, will take the longest to recover and the recovery time will increase as the MAF number increases, where the shortest recovery time will be found for a perturbation on the last MAF (figure 3). We see that for 10% recovery the MAFs are indeed ordered to the recovery time of a perturbation in their direction. For 50% recovery, this is true for the meta-population and the Sahara model but not for the genetic network; and for 90% recovery it is only true for the meta-population model and not for the Sahara model or the genetic network.

The time trajectories of the perturbations are plotted in electronic supplementary material, figures S1 and S2. Here, we see that for the Sahara model the recovery happens in a gradual way, just as in figure 2*e*,*f* in the meta-population model. However, in the genetic network, we see some fluctuations before recovery occurs, a result of the complex eigenvalues of the model, which explains why directional autocorrelation does not reflect recovery times well.

**Figure 4.**
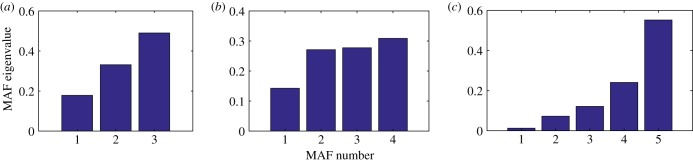
Difference in autocorrelation between MAFs as indicated by their eigenvalues *E*. MAFs are ordered by their eigenvalues *E* (as described in step 3 of the MAF procedure) in the meta-population model (*a*), the Sahara model (*b*) and the genetic network (*c*). Low eigenvalues indicate a high autocorrelation. If two eigenvalues are similar, this indicates that there is only a small difference in the recovery times of the MAFs, for example with MAF 2 and 3 in the Sahara model (*b*), which can be verified in figure 3. (Online version in colour.)

Apart from the recovery times, we also calculate the MAF eigenvalues that indicate how different the autocorrelations on the different MAFs are from each other. Figure 4 shows the MAF eigenvalues for every MAF for the meta-population model (*a*), the Sahara model (*b*) and the genetic network (*c*). For the meta-population model and the genetic network, there is a clear increase in the MAF eigenvalue for increasing MAF number, indicating a clear difference in autocorrelation for the different directions. For the Sahara model, there is hardly any difference in autocorrelation for MAF 2, 3 and 4. This is also reflected in the recovery times of perturbations on MAFs 2, 3 and 4 (figure 3; electronic supplementary material, figure S1).

Last, we perturbed the system in 1000 random directions and calculated the recovery time for all of them. Here, we see that for the non-spiralling systems (the meta-populated and the Sahara model) the first MAF was the direction of slowest recovery. For the spiralling genetic network, however, even though a perturbation on the first MAF yielded a slower recovery than a perturbation on the other MAFs, it was not the slowest direction of the system (electronic supplementary material, figures S3–S5). This shows that, for this model, the direction of maximum autocorrelation is not representative for the direction of slowest recovery. This model is a reactive model [29], where perturbations exist that first grow in amplitude before they return to their equilibrium. These directions affect the MAF analysis. The other two models are not reactive (electronic supplementary material, page 10).

### 3.2. Effect of time-series length

**Figure 5.**
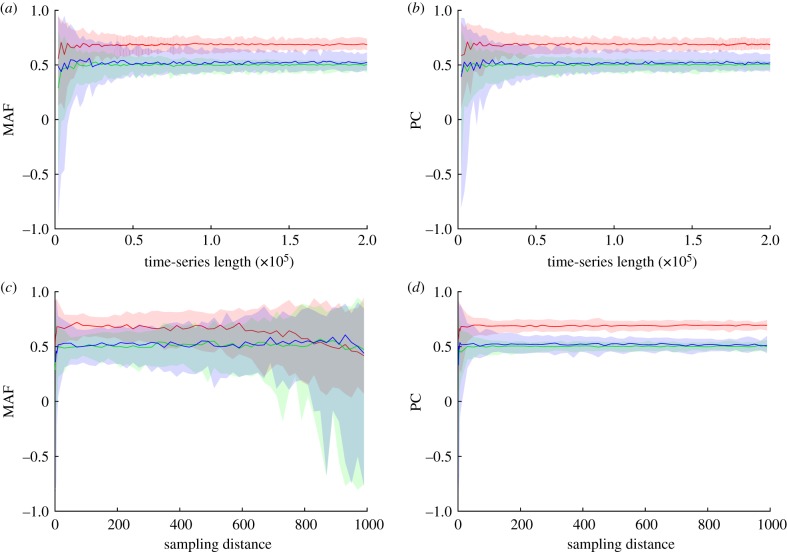
(*a*,*b*) The effect of time-series length on MAF (*a*) and PCA (*b*) for the meta-population model. MAF and PCA are calculated using a block bootstrap with 100 blocks of increasing size (horizontal axis). Solid lines indicate the median of the 100 blocks for every variable. Shaded areas show the 5% and 95% borders. Both MAF and PCA need blocks with a size of about 60 000 to reach an accurate result. (*c*,*d*) The effect of data resolutions on MAF (*c*) and PCA (*d*) for the meta-population model. Again, solid lines show the median of 100 blocks with an increasing distance between consecutive data points (horizontal axis). Shaded areas capture the 5% and 95% borders of the 100 blocks. The analyses clearly show that data resolution has a strong effect on the MAF analysis, whereas it does not affect PCA. (Online version in colour.)

We evaluate the effect of the length of the time series on the robustness of the results by performing our data suitability test, which consists of a block bootstrap with increasing block size. We find that for all our models there is clear convergence for the first MAF and the first PCA, indicating that the data are suitable for the analysis (figure 5*a*,*b* for the meta-population model and electronic supplementary material, figures S6 and S7 for the other two models). MAF and PCA both need about 60 000 time points for the meta-population and Sahara model and 20 000 time points for the genetic network before convergence of the 90% confidence interval is reached.

### 3.3. Effect of data resolution

To evaluate the effect of data resolution, we perform the block bootstrap for different data resolutions (figure 5*c*,*d* for the meta-population model and electronic supplementary material, figures S6 and S7 for the other two models). The first striking observation is that a sampling distance of 1 does not yield the smallest confidence interval, indicating that for both methods it is possible that the data are over-sampled, in which case reducing the amount of data could improve the result. Second, for increasing distance between points, MAF results become inaccurate, whereas data resolution does not affect PCA.

### 3.4. Effect of noise distribution over variables

In our previous analysis, we used Gaussian additive white noise, which is the same for all variables. To evaluate the effect of different noise types, we experiment with differently distributed noise over the different variables. For all analyses, we keep the sum of the noise at 0.2 ($\sum_{i}\delta_{N_{i}}=0.2$).

**Figure 6.**
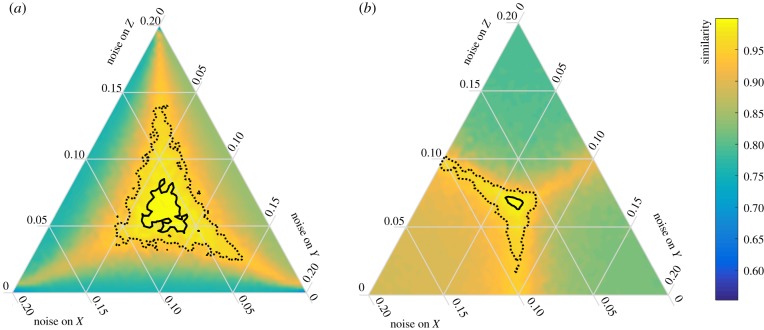
Performance of (*a*) MAF and (*b*) PCA for different noise regimes for the meta-population model with three patches. A location in the plot shows how the noise is distributed over the three variables. In the middle, every variable gets the same amount of noise. In every location, the sum of the three noise levels is 0.2. The performance is calculated by the similarity of the MAF or PCA result to the slowest direction. For noise that is the same for all variables, MAF and PCA give the same (correct) result, as indicated by the high similarity index in the middle of the panels (bright yellow colour). If one or more of the variables receive little or no noise, MAF does not perform well. MAF outperforms PCA in most other cases. The area inside the dotted lines is the area where the similarity is higher than 0.95 (reasonable performance); the area within the black lines is the area where similarity is higher than 0.99 (good performance). (Online version in colour.)

Figure 6 shows the performance of MAF and PCA for different noise regimes. A location in the plot represents the noise distribution over the three variables and the colour scale indicates the performance. Performance is measured as the similarity between the MAF/PCA direction and the direction of slowest recovery. For instance, a similarity of 0.8 means that the probability of finding two random vectors that have an angle that is smaller than the angle between the two vectors is 0.2. For this model, the true direction of slowest recovery is on the vector [0.68  0.52  0.52]. If the result of PCA and MAF point in the direction of only two variables, such as [0.70.70], our similarity measure yields a score of 0.92. Therefore, similarity values lower than 0.95 are not very meaningful. We consider the performance of the method to be ‘reasonable’ when the similarity between the two vectors is higher than 0.95 and ’good’ when similarity is higher than 0.99 (see contours).

Overall, under most noise regimes, MAF performs better than PCA, as indicated by a larger area within the solid and the dotted black lines. Only when noise is low on one variable, and relatively high in the two other variables, does PCA outperform MAF. The reason that PCA works better in that case is that the first PC will point in the direction of the two variables with noise, and this will yield a high similarity score. The same happens for MAF when there is noise on only one variable, in which case the first MAF points to the two variables without noise. The figures show that PCA is only truly meaningful when the noise level is the same for all the involved variables. MAF is a bit more robust and, even when noise levels vary slightly, the method maintains its high accuracy. However, both method fail to obtain the direction of slowest recovery when there is a large difference in noise levels for the different variables.

If the noise becomes larger, the results are not affected (electronic supplementary material, figure S8), assuming that the system remains in the area around its equilibrium. For shorter time series, the accuracy of both methods (MAF and PCA) is reduced (electronic supplementary material, figure S9). The performance of MAF is more affected by data size than the performance of PCA.

## 4. Discussion and conclusion

Our work reveals new ways in which multivariate time series may be mined to detect the direction of lowest resilience in complex systems. Since we are living in a time when more and more high-density data are becoming available [30], new methods to use these data to their full potential are a welcome expansion of the toolbox to analyse complex systems. Our method makes use of the temporal behaviour of the system on small time scales, providing information that is hard to extract from the data by more traditional statistical methods. This also means that the input data have to be sampled at a time interval that is sufficiently small. What exactly is ‘sufficiently small’ depends on the time scale of the dynamics of the system. For instance, brain activity should be measured at much smaller time steps than tree cover. It will typically be difficult to decide *a priori* what sampling frequency and time-series length are appropriate. However, a simple way to test whether or not a particular time series is suitable for the proposed analysis is to run the analyses for different time-series lengths (see Methods, figure 5 and electronic supplementary material, figures S6 and S7). If convergence is reached, and the confidence interval is small, the time series can be considered to be of sufficient length for the proposed analysis.

We showed that both high autocorrelation and high variance in a particular direction in multivariate time series can act as a pointer to the dominant slow direction of a system, provided that the system is not highly reactive and has no strong oscillating dynamics. Importantly, both methods have advantages and disadvantages, so it depends on the available data which method is expected to be most reliable. In one-dimensional systems, autocorrelation is found to be a more robust indicator of resilience than variance [31]. Also in multiple dimensions, we show that autocorrelation outperforms variance when noise levels vary for different variables (figure 6). Intuitively this makes sense, since all variance-based measures such as PCA, covariance and standard deviation are heavily influenced by noise levels. Still, MAF may also lose accuracy when noise only affects a subset of the variables (figure 6). Furthermore, if there is no noise in the slowest direction (i.e. the dominant eigenvector), resilience indicators can miss signals of slow dynamics [32]. Thus, in general, autocorrelation seems more robust than variance. However, the MAF analysis requires a high data resolution to capture the slow dynamics. Resolution is not an issue for PCA, which does not take the timing of the data into account. In conclusion, if the measured variables are known to be subject to different noise levels, MAF should always be preferred. If, however, data are too sparse to get a reliable estimate of a direction with high autocorrelation, PCA might be a good alternative.

There are several caveats when it comes to interpreting the results of our method. First of all, the information we obtain depends on how large the natural fluctuations are (or the noise is). We can only reliably estimate the speed of the system for the part of the state space that is visited by the system. We show that, under some conditions, the local information about slow and fast recovery may be extrapolated somewhat outside this range. However, in real systems, it will typically be impossible to know whether or not this works as we lack complete insight into the properties that shape the dynamics throughout the state space. Another fundamental limitation is the assumption that the system has a stable point attractor. For systems that show oscillating, reactive or chaotic behaviour, the method is not applicable, and more generally the same is true for systems that are far from equilibrium. Also, nonlinear systems or reactive systems often display spiralling dynamics, even if the attractors are stable points (e.g. our gene regulatory network). For these types of systems, PCA will still find a direction of high variance and MAF will still find a direction of high autocorrelation, but these directions do not necessarily correspond to the direction of longest recovery and thus the engineering resilience of the system in that direction. Whether or not a system is expected to fall into this category can be tested based on the time series of the system with an estimation of the ‘worst-case reactivity’ of a system [33]. Also, for systems that have instabilities and that could leave their equilibrium, the direction of MAF or PCA might still point to the direction in which the system will lose its stability. We have deliberately limited ourselves to detecting the mix of perturbations from which the system recovers most slowly. However, the direction of lowest resilience may in some systems also be the direction in which compound perturbations may most easily trigger a critical transition into a new state [34].

Despite these limitations, MAF and PCA offer exciting opportunities to start probing the resilience of multivariate complex systems in novel ways. Our approach builds on the influential work on detecting instabilities based on the phenomenon of critical slowing down in the vicinity of tipping points. Clearly, the phenomena we describe are just the tip of the iceberg when it comes to probing resilience in real systems. Our results show that creative use of known computational tools allows to make the theory of resilience indicators applicable for multivariate systems. The patterns we find suggest ways to move forward to produce theoretical frameworks that help unravel resilience in the wide range of high-dimensional systems on which humanity depends.

## Supplementary Material

Supplementary materials and Figures

## Data accessibility

The code that generates the data and reproduces all the figures can be downloaded from https://github.com/elsweinans/Direction_lowest_resilience
