# Supplementary material for: Finding the direction of lowest resilience in multivariate complex systems
Source: J R Soc Interface. 2019 Oct 30;16(159):20190629. doi: 10.1098/rsif.2019.0629 (PMC6833331; doi:10.1098/rsif.2019.0629)
Supplement: Supplementary materials and Figures [file rsif20190629supp1.pdf]

Finding the direction of lowest resilience in multivariate complex systems

Supplementary materials

Els Weinans, J. Jelle Lever, Sebastian Bathiany, Rick Quax, Jordi Bascompte, Egbert H. van Nes, Marten Scheffer, Ingrid A. van de Leemput

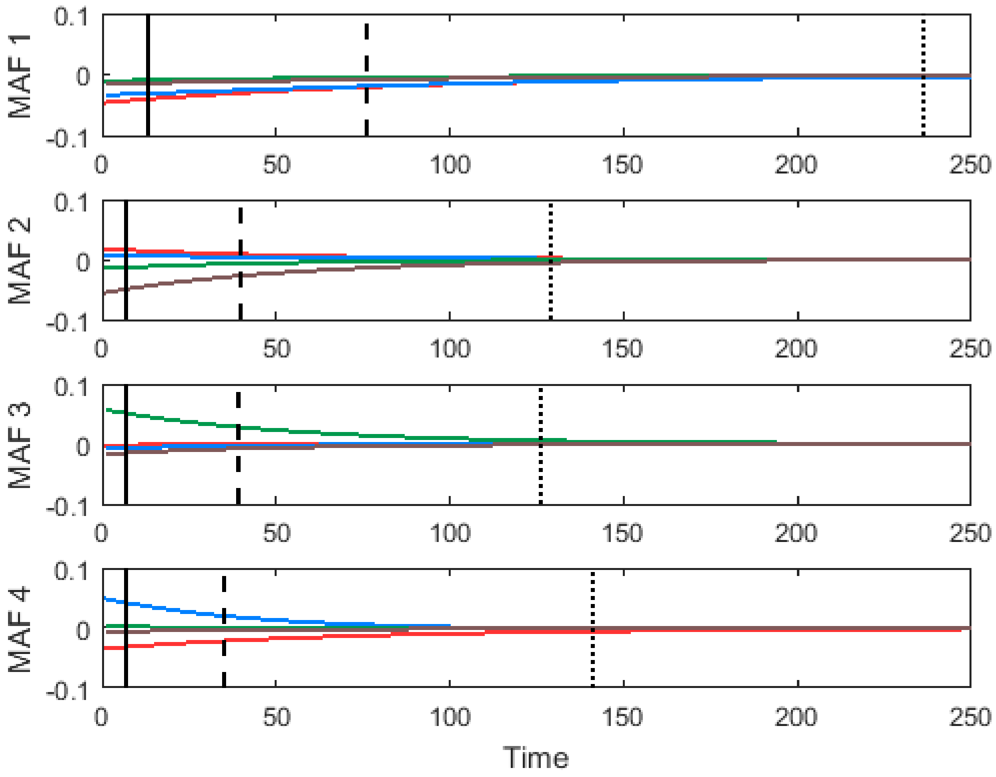

Figure S1: Perturbations on the four MAFS for the Sahara model. 10%, 50% and 90% recovery are indicated with lines, dashed lines and dotted lines respectively. 10% and 50% are well ordered, with no or hardly any difference between the second, third and fourth MAF. 90% Recovery is not well-ordered since the perturbation on MAF 4 results in a higher recovery time. This result is summarized in figure 3.

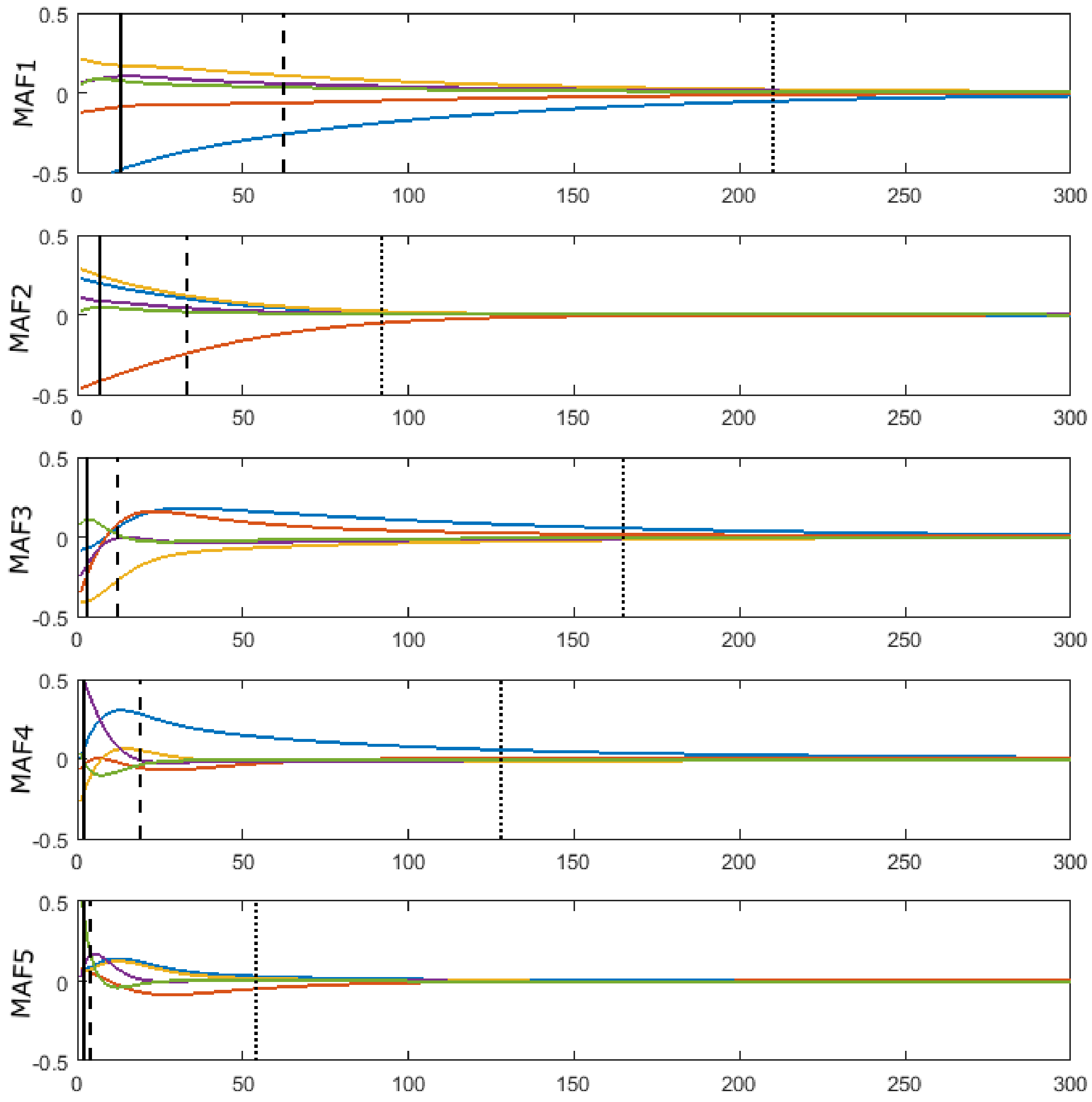

Figure S2: Perturbations on the five MAFs for the genetic network. 10%, 50% and 90% recovery are indicated with lines, dashed lines and dotted lines respectively. 10% recovery is well ordered, 50% and 90% recovery are not well-ordered. The figure clearly shows that the perturbation on MAF 3 ends up on MAF 1 (high deviation for blue and red in the same direction and a small deviation for yellow in the opposite direction). Also, the perturbations on MAF4 and MAF5 clearly show spiralling dynamics, therefore only the initial recovery rate (10% recovery) is well ordered. This result is summarized in figure 3.

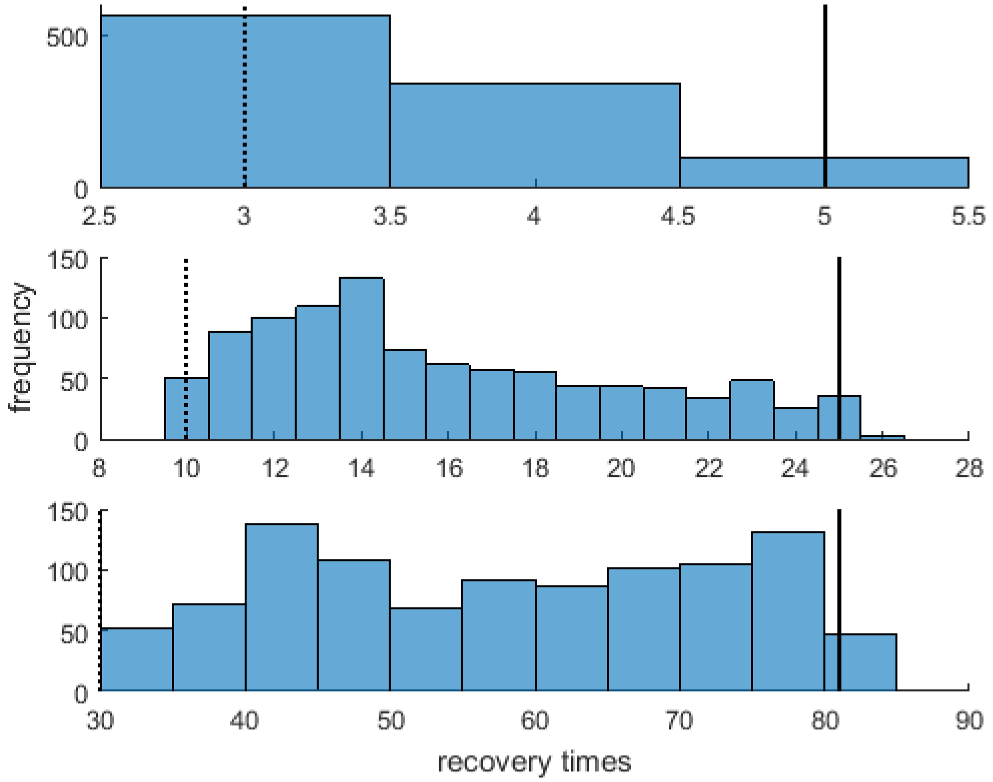

Figure S3: frequency distribution of the recovery times of 1000 perturbations in random directions for 10% (up), 50% (middle) and 90% (bottom) recovery for the meta-population model. Dotted lines indicate the recovery time of a perturbation on the last MAF, solid lines indicate a perturbation on the first MAF. It shows that for the meta-population model MAF finds the direction of slow and of fast recovery.

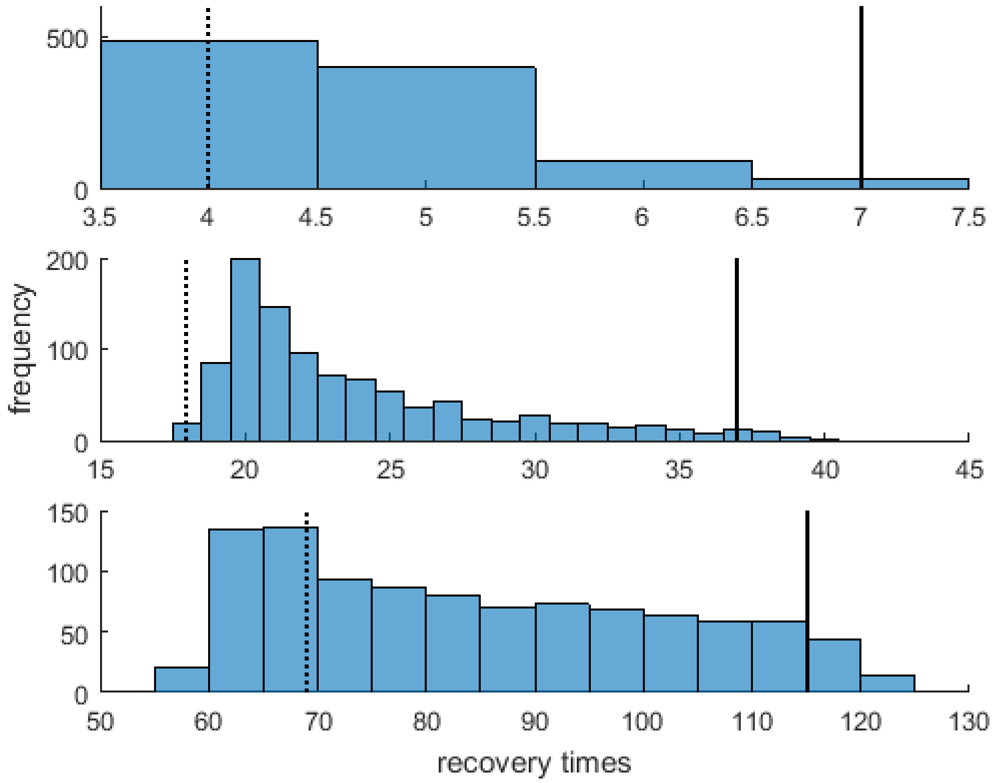

Figure S4: frequency distribution of the recovery times of 1000 perturbations in random directions for 10% (up), 50% (middle) and 90% (bottom) recovery for the Sahara model. Dotted lines indicate the recovery time of a perturbation on the last MAF, solid lines indicate a perturbation on the first MAF. It shows that for the Sahara model MAF finds the direction of slow and of fast recovery.

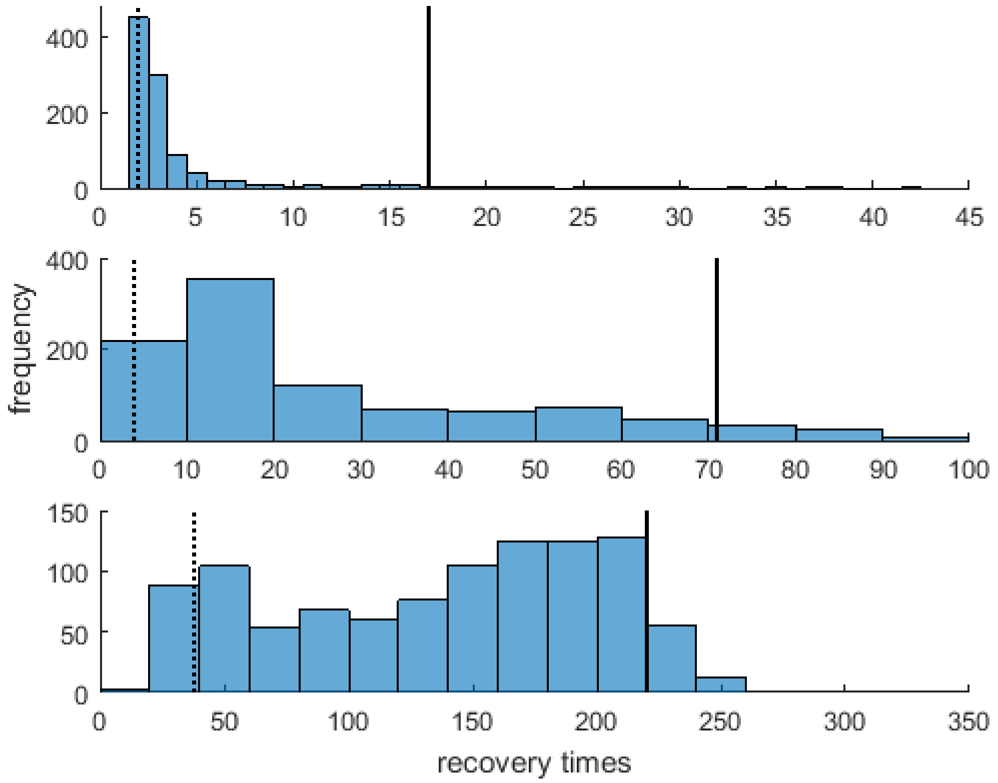

Figure S5: frequency distribution of the recovery times of 1000 perturbations in random directions for 10% (up), 50% (middle) and 90% (bottom) recovery for the genetic network. Dotted lines indicate the recovery time of a perturbation on the last MAF, solid lines indicate a perturbation on the first MAF. It shows that for the genetic network, MAF cannot find the direction of the longest and shortest recovery.

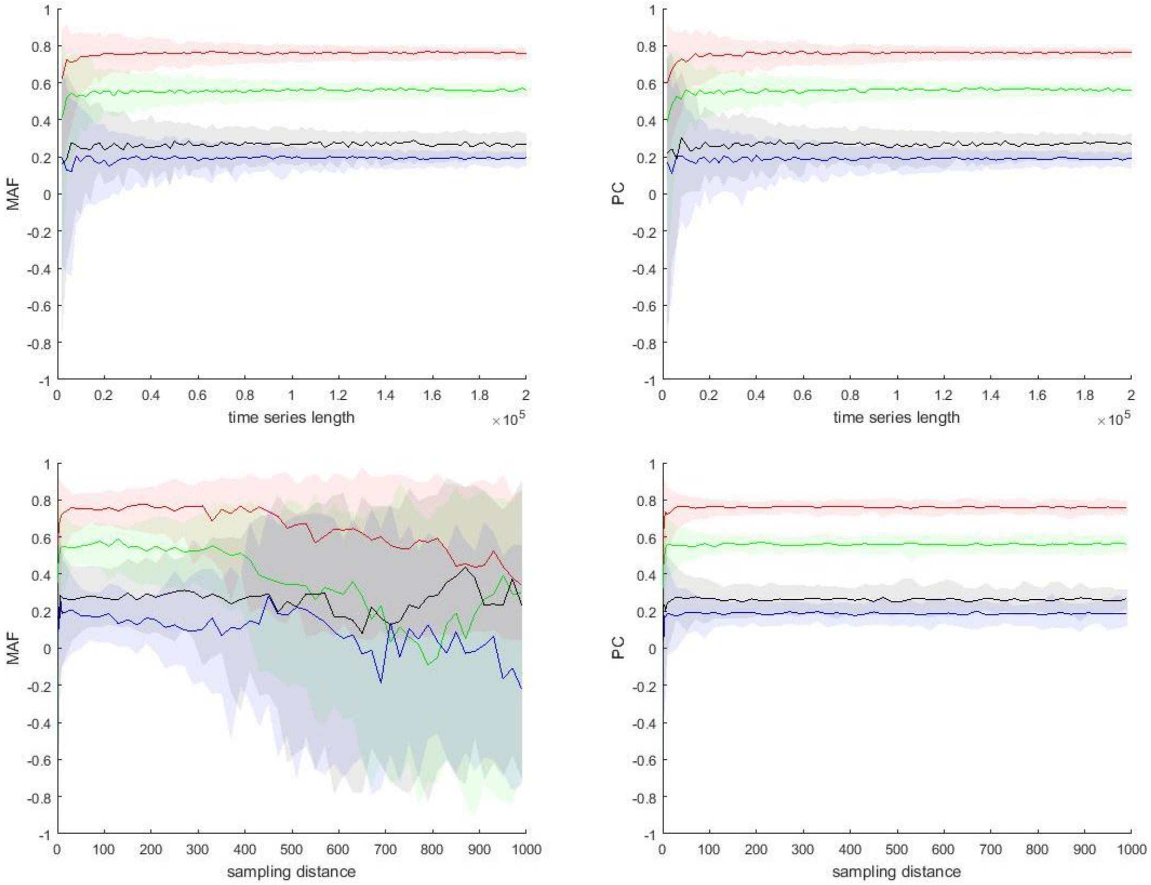

Figure S6: Top row: Effect of time series length on MAF (top left) and PCA (top right) for the Sahara model. Solid lines indicate the medians of 100 blocks in a block bootstrap, shaded areas show the 90% confidence interval. The figures show that both methods improve for increasing data length, but converge at around 100.000 datapoints. Bottom row: Effect of data resolution on MAF (bottom left) and PCA (bottom right) showing that if the distance between datapoints becomes too large, MAF results do not converge anymore, whereas this distance does not affect PCA.

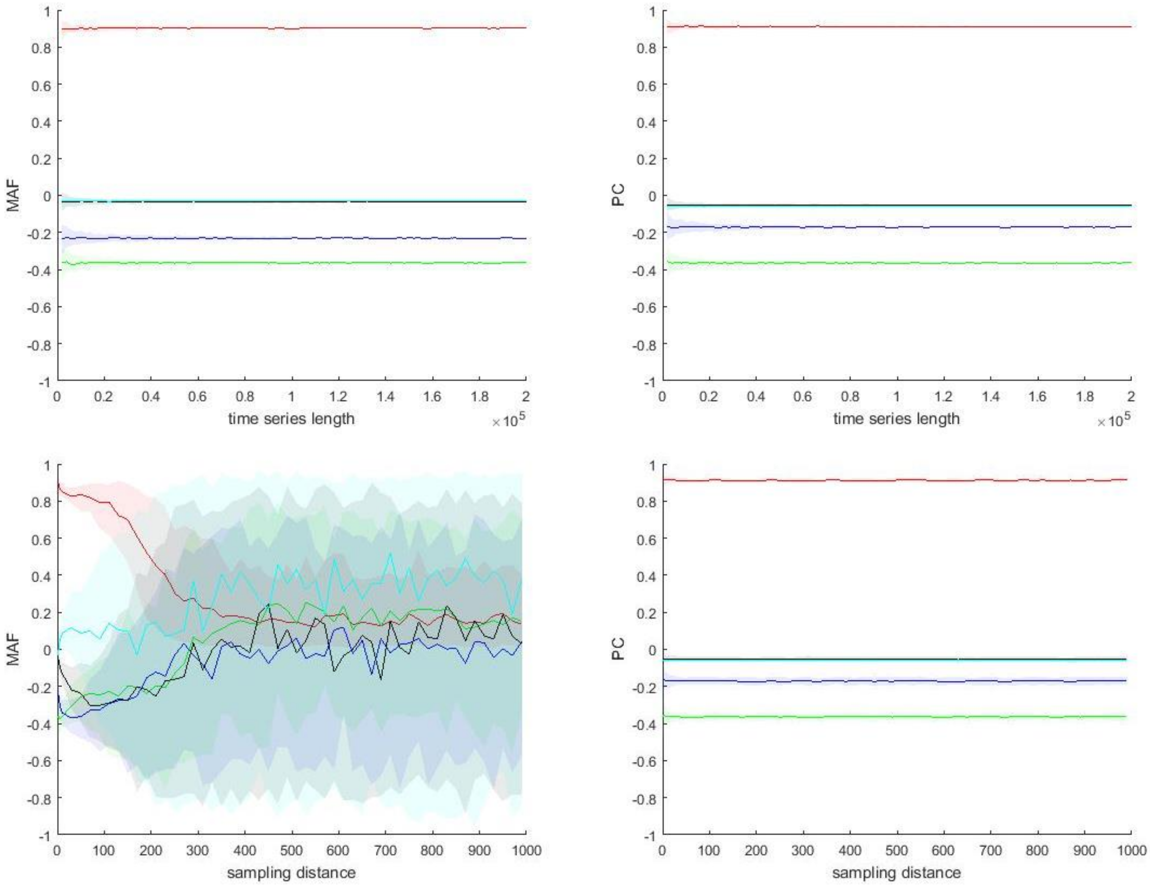

Figure S7: Top row: Effect of time series length on MAF (top left) and PCA (top right) for the genetic network. Solid lines indicate the medians of 100 blocks in a block bootstrap, shaded areas show the 90% confidence interval. The figures show that both methods improve for increasing data length, but converge at around 30.000 datapoints Furthermore, note that when converged, MAF and PCA yield slightly different directions. Bottom row: Effect of data resolution on MAF (bottom left) and PCA (bottom right) showing that if the distance between datapoints becomes too large, MAF results do not converge anymore, whereas this distance does not affect PCA.

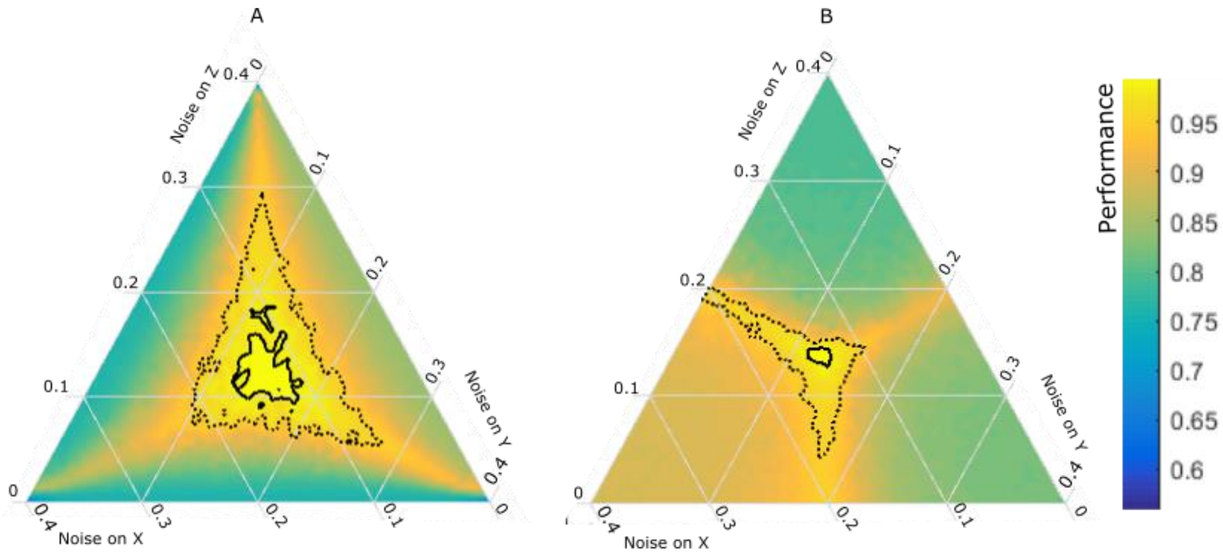

Figure S8: Performance of (a) MAF and (b) PCA for different noise regimes for the meta-population model with three patches. A location in the plot shows how the noise in distributed over the three variables. In the middle, every variable gets the same amount of noise. In every location, the sum of the three noise levels is 0.4, which is twice as high as figure 5. The performance is calculated by the similarity of the MAF or PCA result to the slowest direction. For noise that is the same for all variables, MAF and PCA give the same (correct) result, as indicated with the high similarity index in the middle of the panels (bright yellow colour). If one or more of the variables receive little or no noise, MAF does not perform well. MAF outperforms PCA in most other cases. The area inside the dotted lines is the area where the similarity is higher that 0.95 (reasonable performance), the area within the black lines is the area where similarity is higher than 0.99 (good performance). Comparison with figure 5 indicates that for this model the results remain unchanged for higher noise levels (provided that the noise does not force the system into a new regime). This may be due to the smooth stability landscape of this gradient system.

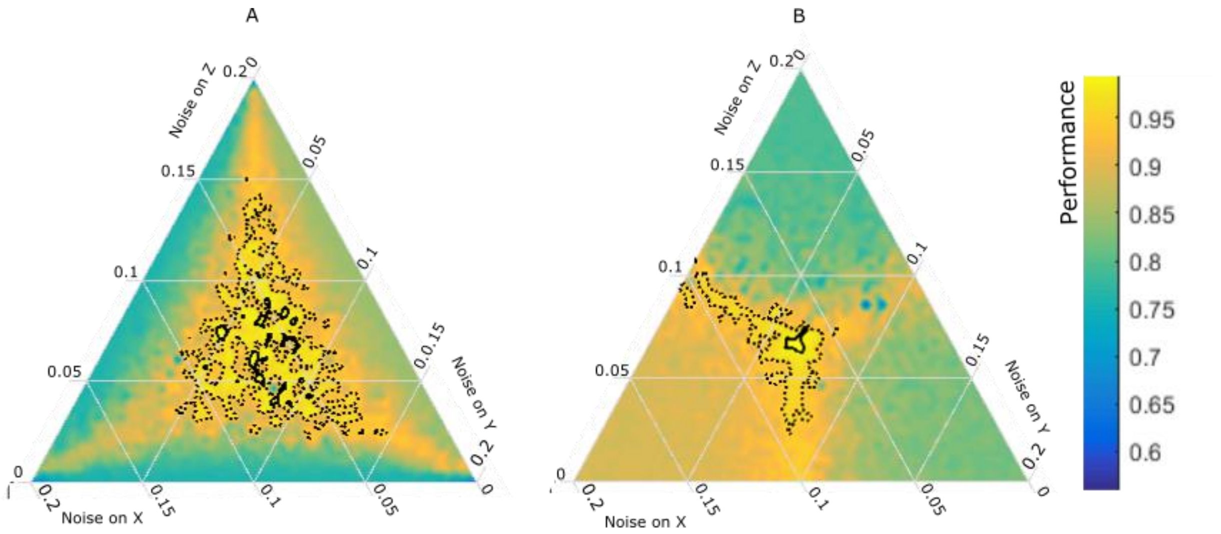

Figure S9: Same as figure S8 but with only 300 data points, as opposed to 4000 as in figure 5 and S8. For equal noise levels over three variables, PCA results are more reliable than MAF results, since there is a clear black circle (99% similarity to true direction of slowest recovery) around the middle of panel B. However, when noise levels vary over variables, both methods become inaccurate, but MAF has a larger region where the performance is at least "reasonable" (95% similarity to true direction of slowest recovery, are within dotted lines).

Gradient systems and reactivity

Gradient systems are systems that have a potential. This means that if you perturb that system in one direction, it will always follow the same path. No spiralling dynamics will occur (i.e. eigenvalues are real). Completely gradient systems are not common in the real world. However, various systems may behave as a gradient system locally around a stable equilibrium. If a system is described by $\frac{dx_i}{dt} = f_i(\vec{x})$, one can evaluate whether or not it is a locally gradient system by checking the equality:

$$\frac{\partial f_i}{\partial x_j} = \frac{\partial f_j}{\partial x_i} :\ i \neq j$$

This means that the Jacobian is symmetric for all possible values for $\vec{x}$. If the jacobian is only symmetric at a stable equilibrium, we say that the system locally has a potential. If this is the case, we expect simple dynamics and autocorrelation should correspond to recovery time. For these type of systems a method such as MAF is most useful.

Autocorrelation still corresponds to recovery time for more complex systems. If the system does not have a potential (i.e. it is not a gradient system), it can still recover from perturbations in a smooth way. However, for certain types of systems there exist directions where a perturbation will amplify before returning to the equilibrium, even though the equilibrium is stable. These systems are called reactive systems.

Whether or not a system is reactive can be tested by comparing the dominant eigenvalue (highest eigenvalue) of the Jacobian to the dominant eigenvalue of the corresponding Hermitian. The Hermitian is calculated as $H = \frac{J+J'}{2}$, where H is the Hermitian and J is the Jacobian. If the eigenvalues for the Jacobian are all negative this indicates a stable equilibrium. If however the Hermitian has also at least one positive eigenvalue, this means that the system is reactive.

For all deterministic models we calculated the Jacobian, the dominant eigenvalue of the Jacobian and the dominant eigenvalue of the corresponding Hermitian in order to evaluate whether or not the system is locally gradient and/or reactive.

Metapopulation model

The Jacobian of the threepatch model is symmetric, indicating that this system locally behaves like a gradient system (actually, this system is a completely gradient system, but we are only interested in local dynamics). Consequently, the dominant eigenvalue of the Jacobian (-0.2851) is the same as the dominant eigenvalue of the Hermitian (-0.2851) indicating no reactivity. Therefore this is a system where autocorrelation is expected to correspond to recovery time.

Sahara model

The Jacobian of the Sahara model is not symmetric and therefore this is not a gradient system. The dominant eigenvalue of the Jacobian (-0.5223) is different from the dominant eigenvalue of the Hermitian (-0.4621), but since both eigenvalues are negative this should not lead to reactive behaviour and autocorrelation is expected to correspond to recovery time.

Genetic network

The Jacobian of the genetic network is not symmetric and therefore this is not a gradient system. The dominant eigenvalue of the Jacobian (-10.50) differs considerably from the eigenvalue of the Hermitian (10.99). Furthermore, even though the dominant eigenvalue of the Jacobian is negative, corresponding to a stable equilibrium, the dominant eigenvalue of the Hermitian is positive, which means that it is possible that some perturbations grow initially even though the equilibrium is stable. In these directions, perturbations will take a long time to recover even though the autocorrelation in this direction is not necessarily high. Consequently, autocorrelation in these systems is not a good indicator for recovery time.
